# Effective models for antimetastatic therapies

**DOI:** 10.18632/oncotarget.18379

**Published:** 2017-06-06

**Authors:** Lin Tian, Xiang H.F. Zhang, Sendurai A. Mani

**Affiliations:** Xiang H.F. Zhang: Lester and Sue Smith Breast Center, Dan L. Duncan Cancer Center, Department of Molecular and Cellular Biology, McNair Medical Institute, Baylor College of Medicine, One Baylor Plaza, Houston, Texas, USA; Sendurai A. Mani: Department of Translational Molecular Pathology and Metastasis Research Center, MD Anderson Cancer Center, Houston, Texas, USA

**Keywords:** EMT, metastasis, breast cancer, murine models, tumor immunology

Despite the successful therapeutic regimens for the treatment of primary tumors, effective interventions for metastatic lesions are lacking. Metastasis is a complex process, wherein the cancer cells pass through multifaceted steps from the primary site to establish tumor at the distant site. This multifaceted process begins with the establishment of highly vascularized primary tumor, local invasion, intravasation, survival in the circulation and the establishment of secondary tumor or metastasis at the distant site. In addition to the intricate nature of the metastasis, this process remains the primary cause of most cancer-associated death [1-2]. We have previously shown that the epithelial-mesenchymal transition (EMT) play a vital role in promoting metastasis from the mammary fat pad for breast cancer to distant organ and remain as a one of the rate limiting step for metastasis [3-5]. We also demonstrated that the cancer cells not only become migratory and invasive but also acquire stem cell properties by activating the EMT program, which could help the cancer cells face the harsh environment at various stages of metastatic progression and assist the cancer cells in establishing a tumor similar to the primary tumor at the secondary site [6].

One of the primary reason for the lack of therapies is poor understanding the biology of this process. In particular, lack of proper experimental models to study this extremely complicated metastatic process including disseminating cancer cells from primary tumors and subsequently forming colonization in distant organs. Moreover, the majority of the studies involves the introduction of human cancer cell lines via tail vein or intracardiac route of highly immunocompromised mice to establish metastasis at the distant organ such as lung, bone, and brain to study their growth. While these models yielded valuable information, it never addressed the role of invasion and intravasation. Most importantly, these animal models with severe deficiency of adaptive immune systems completely ignore the effects of immune surveillance. Therefore, the preclinical models, which develop metastasis from the orthotopic site such as a mammary fat pad for breast cancer in an animal with the intact immune system is needed to investigate the biological mechanisms of metastasis and to develop therapeutic interventions for this lethal metastatic process.

In a recent issue of Oncotarget [7], Dr. Wakefield group characterized a series of metastatic xenografts in an immune intact animal from the mammary fat pad to various organs. To mimic the clinical setting, the authors used a panel of 12 transplantable murine breast tumor cell line models and measured spontaneous metastasis following primary tumor resection. These murine breast tumor models display a variety of phenotypes, which captures the human breast tumor heterogeneity. At the molecular level, the authors assessed the relationship of murine breast tumor panel to human breast tumor by exome sequencing. Indeed, many of the top 30 most frequent mutations in human breast cancer are also found in these murine breast tumor models.

**Figure 1:**
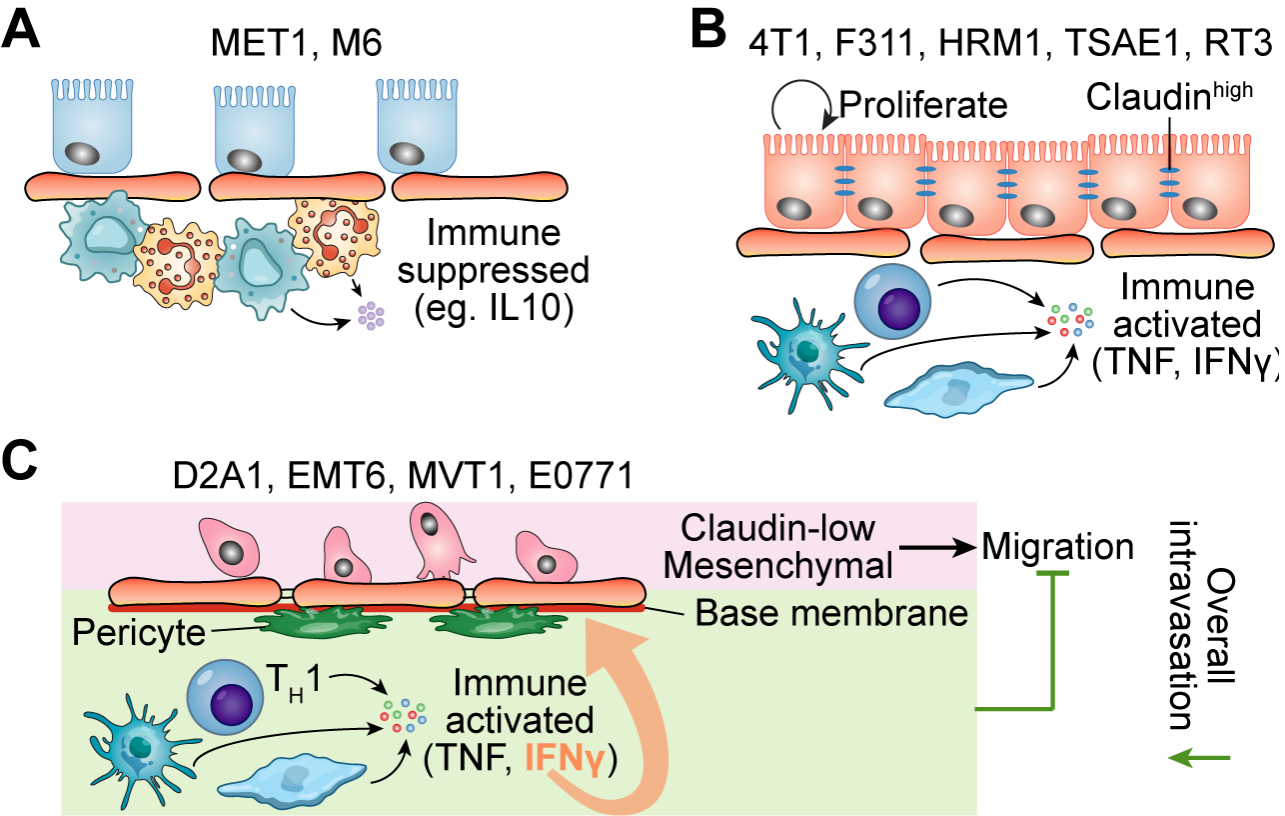
Murine tumors in different clustering groups showing different immune signatures and claudin expressions **A.** The models in Cluster I had immunosuppressive signatures such as high expression of IL10RA and low expression of TNF and IFNγ. **B-C.** In addition to immune signatures, the claudin expression level can be used to further distinguish tumor models in Cluster II and Cluster III. B, The models in Cluster II demonstrated high proliferation signature. C, The models in Cluster III exhibited low differentiation and mesenchymal signatures.

It is worth noting that the cell lines from spontaneous tumors have significantly higher number of SNV burden than the cell lines derived from genetically engineered mouse models (GEMMs). At a phenotypic level, the authors quantified the patterns of proliferation, apoptosis, angiogenesis and immune cell infiltration. Microarray transcriptomic profiling revealed the underlying biological pathways that were associated with the different phenotypes. Interestingly, MET1, and M6 had low IFNγ expression and displayed immunosuppressive signatures (Figure 1A). The rest of the models can be further divided into two groups according to *Claudin* expression. The *Claudin*-high models (4T1, F311, HRM1, TSAE1, R3T) displayed higher activities in pathways including proliferation, angiogenesis, and estrogen pathway than the *Claudin*-low models (D2A1, EMT6, MVT1, E0771) (Figure 1B). One model, E0771, has several interesting features: 1) the highest mutation load (5 times greater than the average across all the models); 2) striking necrotic sites in E0771 showed massive immune cell infiltration. Considering E0771 has low expression of *Claudin* gene and is mesenchymal-like, this raises a question why E0771 has low ability to develop metastasis and low invasion/migration signature. A recent paper used E0771 as the model to study the role of the adaptive immune system in tumor vascular normalization may provide the answer [8]. Despite the rare lung metastasis in immunocompetent mice, the mice lacking CD4^+^ T lymphocytes displayed substantially high number of circulating tumor cells and frequent lung metastasis after tumor resection. One possible mechanism is IFNγ secreted by Type 1 helper T cells (T_H_1) keep vessel integrity by upregulating adhesion molecules and extracellular matrix gene expression in tumor vessels (Figure 1C), thereby inhibiting E0771 intravasation and subsequently pulmonary metastasis [8].

While the study by Yang et al. not only utilizes the orthotopic tumor model but also used immunocompetent mice and addressed an important gap in our choice of murine tumor models for metastatic studies [7], it also raised multiple important questions relating to the role of immune cells and the local tumor microenvironment. It will be important to perform more comprehensive immunophenotyping and to investigate the immune cell localization pattern in more details. In addition to the quantity, the authors found an interesting pattern of leukocyte spatial distribution: leukocytes are distributed within the tumor in some models while staying around the tumor in the others. It will also be useful to further investigate the localization of certain immune cell population relative to other immune subsets or other stromal components such as endothelial cells and fibroblasts. By addressing these questions, we can make better choices of these models to delineate the tumor heterogeneity and combat metastasis. Finally, and most importantly, it is imperative to use either immune reconstituted human tumor models or syngeneic tumor models to study metastasis, in particular by introducing tumor cells at the orthotopic site, when possible. This would allow us to identify novel opportunities presented by various components of tumor microenvironment including immune cells to combat cancer.
